# Supplementary material for: Systemic Metabolic Alterations Correlate with Islet-Level Prostaglandin E2 Production and Signaling Mechanisms That Predict β-Cell Dysfunction in a Mouse Model of Type 2 Diabetes
Source: Metabolites. 2021 Jan 16;11(1):58. doi: 10.3390/metabo11010058 (PMC7830513; doi:10.3390/metabo11010058)
Supplement: Supplementary file 1 [file metabolites-11-00058-s001.zip › Supplemental Figure 1.docx]

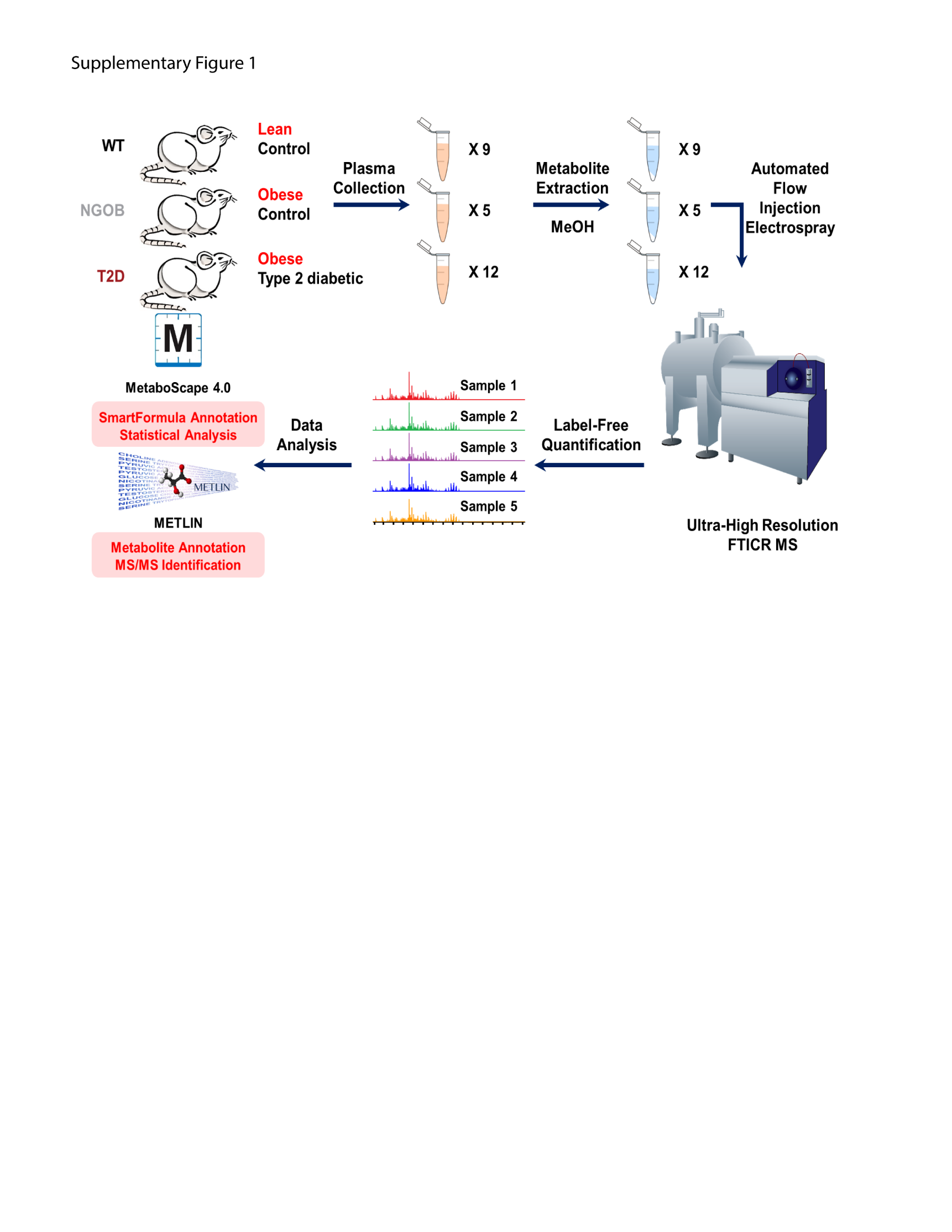


**Supplementary Figure 1:** Workflow of the FIE-FTICR MS-based platform for metabolomics. Plasma samples were first extracted and then directly injected into FTICR MS without LC separation for detection and label-free quantification of metabolite features. Metaboscape 4.0 was used for statistical analysis and SmartFormula annotation. METLIN annotation provided chemical names of putative metabolites by accurate mass matching.
